# The cephalopod arm crown: appendage formation and differentiation in the Hawaiian bobtail squid *Euprymna scolopes*

**DOI:** 10.1186/s12983-016-0175-8

**Published:** 2016-09-29

**Authors:** Marie-Therese Nödl, Alexandra Kerbl, Manfred G. Walzl, Gerd B. Müller, Heinz Gert de Couet

**Affiliations:** 1 Department of Theoretical Biology, University of Vienna, Althanstrasse 14, 1090 Vienna, Austria; 2 Marine Biology Section - Department of Biology, University of Copenhagen, Universitetsparken 4, 2100 Copenhagen, Denmark; 3 Department of Integrative Zoology, University of Vienna, Althanstrasse 14, 1090 Vienna, Austria; 4 Department of Biology, University of Hawaii at Manoa, 2538 McCarthy Mall, Edmondson Hall 413, Honolulu, HI 96822 USA

**Keywords:** Cephalopod, *Euprymna scolopes*, Bobtail squid, Lophotrochozoa, Arm crown, Appendage, Evolution, Development, Tentacle

## Abstract

**Background:**

Cephalopods are a highly derived class of molluscs that adapted their body plan to a more active and predatory lifestyle. One intriguing adaptation is the modification of the ventral foot to form a bilaterally symmetric arm crown, which constitutes a true morphological novelty in evolution. In addition, this structure shows many diversifications within the class of cephalopods and therefore offers an interesting opportunity to study the molecular underpinnings of the emergence of phenotypic novelties and their diversification. Here we use the sepiolid *Euprymna scolopes* as a model to study the formation and differentiation of the decabrachian arm crown, which consists of four pairs of sessile arms and one pair of retractile tentacles. We provide a detailed description of arm crown formation in order to understand the basic morphology and the developmental dynamics of this structure.

**Results:**

We show that the morphological formation of the cephalopod appendages occurs during distinct phases, including outgrowth, elongation, and tissue differentiation. Early outgrowth is characterized by uniform cell proliferation, while the elongation of the appendages initiates tissue differentiation. The latter progresses in a gradient from proximal to distal, whereas cell proliferation becomes restricted to the distal-most end of the arm. Differences in the formation of arms and tentacles exist, with the tentacles showing an expedite growth rate and higher complexity at younger stages.

**Conclusion:**

The early outgrowth and differentiation of the *E. scolopes* arm crown shows similarities to the related, yet derived cephalopod *Octopus vulgaris*. Parallels in the growth and differentiation of appendages seem to exist throughout the animal kingdom, raising the question of whether these similarities reflect a recruitment of similar molecular patterning pathways.

**Electronic supplementary material:**

The online version of this article (doi:10.1186/s12983-016-0175-8) contains supplementary material, which is available to authorized users.

## Background

Cephalopods represent a highly derived and very successful class within the phylum Mollusca, showing adaptations to all marine ecosystems from the deep sea to marine estuaries. They are thought to have evolved from a limpet-like monoplacophoran ancestor during the late Cambrian about 500 million years ago [1]. The eventual transition from a shell-bearing, bottom dwelling organism to a free-swimming, active predator was accompanied by the appearance of a series of features that cannot be found in any other molluscan class and are therefore considered morphological novelties [2]. One of the most intriguing innovations is the evolution of the cephalopod arm crown, which is thought to have either derived in part [3, 4] or entirely [5–7] from the foot of molluscan ancestors. Due to its capacity to enable predatory life styles, it qualifies as a “key innovation” in cephalopod diversification [8].

**Fig. 1:**
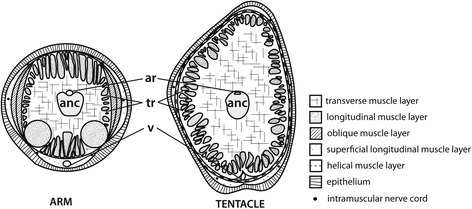
Schematic illustration of transverse sections through an adult squid’s arm and tentacle. anc, axial nerve cord; ar, artery; o, oblique muscle; tr, trabeculae; v, vein; after Kier [16]

The arm crown of modern cephalopods (coleoids) is a bilaterally symmetric structure, consisting of four pairs of prehensile arms with an additional pair of retractable cirri in *Vampyroteuthis* and extensible tentacles in the decabrachian cephalopods. The homologies of the arms in the cephalopod orders have not been definitively resolved. In a study examining a more ancestral, shell-bearing cephalopod, *Nautilus pompilius*, Shigeno et al. [9] have shown that five distinct pairs of arm fields are formed during embryonic development, which give rise to part of the head complex and a multitude of digital tentacles. Despite the differences in the adult structure of nautiloid and coleoid appendages, it seems therefore likely that five pairs of arm were already present in a common ancestor. Studies based on anatomical and embryological comparisons suggest that the second arm pair was then lost in the octobrachian cephalopods and modified in *Vampyroteuthis* [7, 9–12]. In the decabrachian lineage, however, presumably the fourth arm pair was modified into retractile tentacles and optimized for prey capture (Additional file 1). Individual arms and tentacles of the decabrachian arm crown are composed of a dense three-dimensional array of muscle fibers, connective tissue and a central axial nerve cord. These structures were termed muscular hydrostats by Kier and Smith [13] because their musculature serves a dual purpose of providing the appendage with skeletal support and the force for movement. The motor control for the arm’s musculature and suckers is provided by the axial nerve cord, which comprises the largest component of the peripheral nervous system [14, 15]. Despite the similarities in the gross anatomy of arms and tentacles, significant differences in form and function exist, which have been comprehensively studied in a number of decabrachian species [13, 16–19] (Fig. 1).

In particular, the tapered, sessile arms are equipped with suckers from the base to their distal tip and are used for a variety of tasks including prey handling, behavioral display, locomotion and reproduction [20]. The arm’s central axial nerve cord consists of a series of ganglia, each corresponding to one sucker on the oral side of the arm. A transverse muscle layer surrounds the central nerve cord and is positioned perpendicular to the long axis of the arm. It is located adjacent to the longitudinal muscle layer and interdigitates with bundles thereof, forming so-called trabeculae. Two layers of obliquely oriented musculature enclose the longitudinal muscle layer and are each surrounded by one oral and two lateral layers of superficial longitudinal musculature. The latter incorporate six intramuscular nerve fibers, that are connected to the axial nerve cord by connective fibers and to each other by anastomoses [15]. The arm is covered by a loose connective tissue dermis and is enclosed by a simple cuboidal epithelium. This combination of musculature is specifically adapted to the bending movement and torsion of the manipulative and inextensible arm [17].

In contrast, the decabrachian cylindrical tentacles are specialized structures, which are mostly optimized for prey capture. Contrary to the arms, tentacle suckers are only present on their distal club, and associated ganglionic structures as well as most neuronal cell bodies are restricted to this area. Similar to the arms, a large transverse muscle layer surrounds the tentacle’s axial nerve cord. However, an additional layer of circular musculature outlines the adjacent longitudinal muscle fibers. Next to the circular musculature, two thin layers of helical muscle tissue border a superficial longitudinal muscle layer, which incorporates the intramuscular nerve cords. As with the arm, the tentacle’s musculature is covered in a loose connective tissue dermis and is surrounded by a simple cuboidal epithelium [17–19, 21].

As an evolutionary novelty with such diversity the cephalopod arm crown offers an interesting opportunity to address the molecular underpinnings of a number of fundamental evolutionary problems. These include (i) which key changes in gene regulation are associated with the emergence of morphological novelties and (ii) to the diversification of serially homologous structures respectively, as well as (iii) whether shared molecular mechanisms in appendage patterning exist throughout the animal kingdom. The latter has recently been addressed on a morphological level from the standpoint of a more derived cephalopod, the octopus. Nödl et al. [22] have shown surprising similarities in the mechanisms by which appendages are formed in octopus and known model organisms. These similarities include uniform cell proliferation during early arm outgrowth, an elongation along the proximal-distal (PD) axis driven by cell shape changes, and a switch to a progressive, distal growth pattern during tissue differentiation. Considering the presumed evolutionary origin of the arm crown these results are specifically intriguing and raise the question whether the re-organization of the molluskan foot into the cephalopod arm crown has been accompanied by the recruitment of genes known to be involved in appendage formation in vertebrates and insects.

In the past years the Hawaiian bobtail squid *Euprymna scolopes* has become an important model for cephalopod body plan evolution in general and appendage formation in particular [23, 24]. The groundwork for molecular and developmental laboratory experiments has been set and successfully applied [23–29]. Despite increased interest in *E. scolopes* as a developmental model for the cephalopod arm crown innovation, no morphological description of the embryonic formation of this structure exists. However, for the interpretation of gene expression data it is absolutely crucial to understand the basic morphology and the developmental dynamics of the structure under study. In addition, the comparison of the formation of a decabrachian arm crown to that of an octobrachian may shed light onto the evolutionary origin of this structure and its diversification.

In this study we provide a detailed description of the embryonic development and differentiation of the *E. scolopes* arm crown. We investigate the different phases of its development and the similarities with the dynamics observed in octopus. This detailed description of arm and tentacle morphology and development intends to provide a basis for further studies on *E. scolopes* appendage development.

## Results

### *E. scolopes* general development and axes denomination

**Fig. 2:**
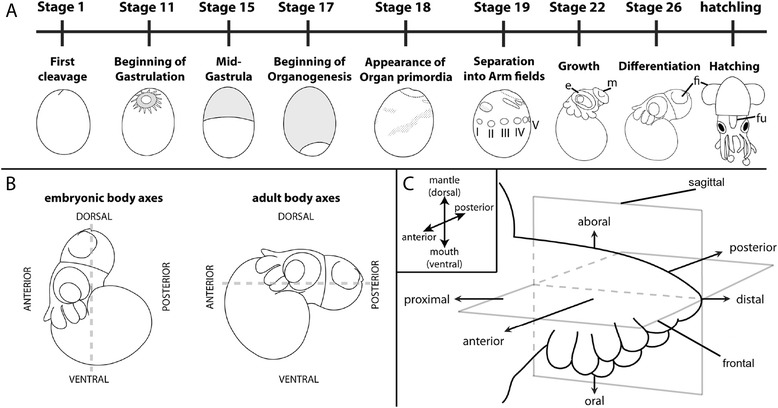
*Eurpymna scolopes* embryonic development and denomination of axes. **a** Schematic overview of *E. scolopes* normal embryonic development after Lee et al. [25]. Development takes about 21 days at 24 °C water temperature. Except for the hatchling, embryos are oriented with dorsal to the top and anterior to the left. The hatchling is shown in a posterior view, which in the physiological orientation of the adult will become the ventral side of the animal. **b** Embryonic (anteroposterior, AP) versus adult (dorsoventral, DV) body axes. As opposed to the physiological orientation of the adult animal, the mantle is considered dorsal and the mouth ventral during embryonic development, while the future dorsal side is considered anterior and the future ventral side posterior in the embryo. **c** Terminology of the embryonic arm’s spatial organization with respect to the embryonic body axes (inset) used in this study. Distal is defined as the tip, proximal as the base of the appendage, the side bearing suckers is considered as oral and the opposite side, as aboral. The side facing the early mouth primordium is regarded as anterior and the one facing the funnel as posterior. Grey rectangles indicate the position of the sections through the arms shown in this study (frontal and sagittal). I – V denotes the arm pairs in the order they are spatially positioned along the AP axis; e, eye; fu, funnel; fi, fin; m, mantle

*E. scolopes* develops by bilateral cleavage, typical for decabrachian cephalopods. Development takes about 21 days at 24 °C water temperature and can be divided into 30 distinct stages, as described by Lee et al. [25] (based on Arnold [30]) and are summarized in Fig. 2a. Cleavage is superficial and leads to a discoblastula. During epibolic gastrulation a thin sheet of cells expands over the yolk, forming the outer yolk sac, while the embryo proper develops at the animal pole of the egg. Shortly before the entire yolk is covered by the yolk sac, organ primordia become visible as epithelial thickenings. These increase in size and complexity until the fully developed paralarva hatches resembling a miniature adult. As usual for cephalopods, the embryonic dorso-ventral (DV) body axis is designated corresponding to the embryo’s orientation along the animal-vegetal axis of the egg. Accordingly, the area of the mouth primordium is regarded as anterior while the area of the anus marks the posterior side of the embryo. During late stage development the body axes tilt by 90° relative to the embryonic axes, so that the original dorso-ventral (DV) axis becomes the antero-posterior (AP) axis (Fig. 2b). This tilted orientation of the animal corresponds to its physiological swim position in the water as an adult.

Regarding the axes of the arms the following terms will be used to describe their orientation: “proximal” will be considered the base of the arm, closest to the animal’s body, “distal” will appropriately refer to the tip of the arm. “Anterior” and “posterior” will correspond to the embryonic anterior (facing the embryonic mouth) - posterior (facing the embryonic funnel) axis. The side of the arm covered in suckers and facing the central adult mouth will be denoted oral while the opposing side will be referred to as aboral (Fig. 2c). The following in depth description of arm bud morphology during embryonic development focuses on the growth and differentiation events of the arm pairs II and IV. The latter develop into the specialized prehensile tentacles, which morphologically set them apart from the rest of the arm pairs. Arm pair II was chosen exemplarily in order to provide continuity of description. The position of the sections through the arms shown in this study are indicated in Fig. 2c.

### Appearance of the arm crown and early arm outgrowth

**Fig. 3:**
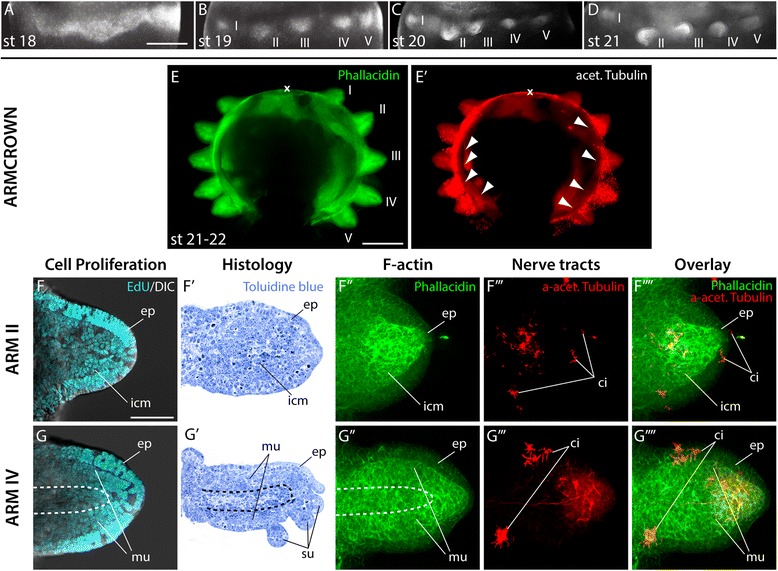
Appearance of the arm crown and early arm outgrowth. (**A**-**D**) overview of *E. scolopes* arm crown development from stages 18 to 21. Arm crowns are either labeled with anti-Histone H1 to visualize cell nuclei (**A**) or phallacidin to visualize F-actin (**B**-**D**), and oriented with anterior to the left and dorsal to the top. (**E**-**E′**) oral view of arm crowns at stage 21–22 labeled with phallacidin to visualize F-actin in green (**E**) and anti-acetylated tubulin to visualize nerve tracts in red (**E′**). (**F**, **G**) confocal image stacks of frontal sections of arm II (**F**) and arm IV (**G**) treated with EdU to visualize proliferating cell nuclei in cyan merged with a DIC image of the arms in the same focal plane. (**F′**, **G′**) frontal (**F′**) and sagittal (**G′**) histological sections of arms stained with toluidine blue. (**F′′**- **G′′′**) confocal image stacks of arm II (**F′′**- **F′′′′**) and arm IV (**G′′**- **G′′′′**) labeled with phallacidin to visualize F-actin in green (**F′′**, **G′′**), anti-acetylated tubulin to visualize nerve tracts in red (**F′′′**, **G′′′**) and their overlap in merged images (**F′′′′**, **G′′′′**). x marks the position of the mouth, I – V denotes the arm pairs in the order they are spatially positioned. Arms are oriented with aboral to the top and distal to the left. White arrowheads in (**E′**) point at the proximal part of the interbrachial ganglia’s axonal tracts joining to form the interbrachial connective. Dashed line in (**G**-**G′′**) marks the area of the axial nerve chord. ep, epithelium; mu, musculature; icm, inner cell mass. Scale bars: 50 μm in (**A**), 100 μm in (**E**)

The arm crown is first recognizable at stage 18 as two continuous bands of cells around the equator of the egg (Figs. 2a, 3A). At stage 19, the arm crown separates into five distinct arm fields consisting of condensed layers of epithelial cells on each side of the embryo (Figs. 2a, 3B, Additional file 2), which quickly increase in size during the following stages of development (Figs. 2a, 3C-D). Arm fields II and IV grow out first, followed by III and V, while arm field I extends last and remains the smallest until the animal hatches. At stage 22, the entire embryo starts to contract, which leads to a rearrangement of all organs to a more definitive state [7, 15]. This whole body contraction moves arm pair I closer together and separates the outer yolk from the smaller inner yolk sac (Fig. 2a stage 19 and 22; 2a, 3E). At this stage, axon tracts of the axial nerve cord are visible at the base of all arm buds and connect to form the interbrachial connective (Fig. 3E′, arrowheads). Individual arms are apparent as epithelial bulges, which show uniform cell proliferation (Fig. 3F, G). While arm bud II consists of an inner cell mass, which in the histological sections shows no apparent differentiation or regionalization surrounded by an epithelium (Fig. 3F′), the inner cell mass of arm IV is made up by a dense outer layer of cells with elongated nuclei (region of future musculature), which surrounds a loose inner layer of cells with spherical nuclei (region of future axial nerve cord; Fig. 3G′). In both cases, the epithelium is comprised of multiple cell layers except at the distal end, where a monolayer of epithelial cells covers a slightly pointed tip (Fig. 3F′- F″, G'- G″). Several ciliated cells become apparent on the aboral surface of the epithelium of both arm pairs II and IV (Fig. 3F'''-F'''', 3G'''-3G''''). Even though a neuropil cannot be detected in histological sections, individual patches of nerve fibers projecting from clusters of neurons towards the proximal base of the arm are visible in arm II (Fig. 3F'''-F''''). In contrast, a small central neuropil region of the forming axial nerve cord is detectable in arm IV, where axon tracts terminate diffusely in an epithelial region just before the distal tip of the arm (Fig. 3G', dashed line; 3G'''-3G'''').

### Elongation along the PD axis

**Fig. 4:**
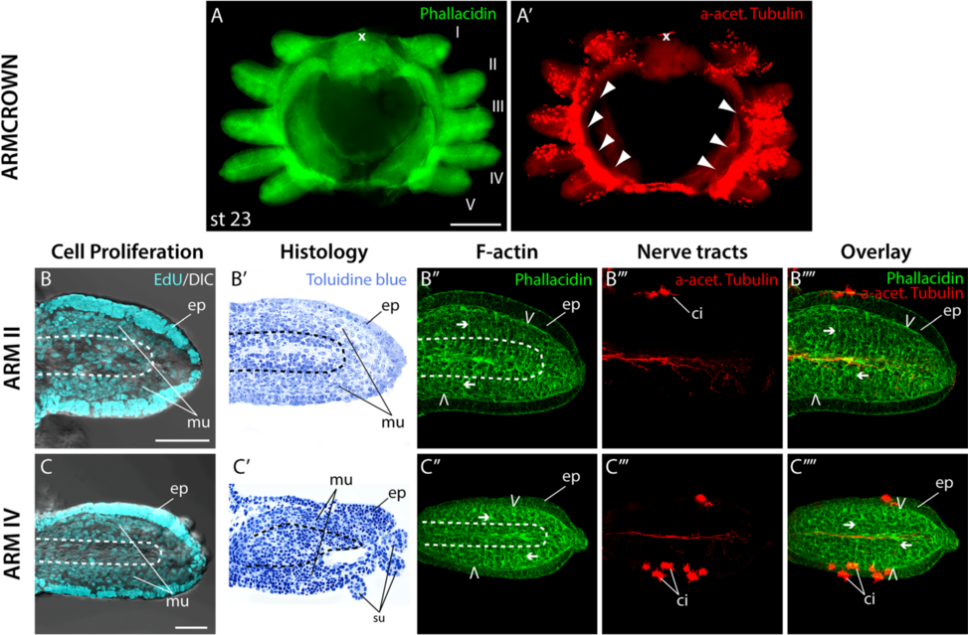
Arm elongation. (**A**-**A′**) oral view of arm crowns at stage 23 labeled with phallacidin to visualize F-actin in green (**A**) and anti-acetylated tubulin to visualize nerve tracts in red (**A′**). (**B**, **C**) confocal image stacks of frontal sections of arm II (**B**) and arm IV (**C**) treated with EdU to visualize proliferating cell nuclei in cyan, merged with a DIC image of the arms in the same focal plane. (**B′**, **C′**) frontal (**B′**) and sagittal (**C′**) histological sections of arms stained with toluidine blue. (**B′′**- **C′′′**) confocal image stacks of arm II (**B′′**- **B′′′′**) and arm IV (**C′′**- **C′′′′**) labeled with phallacidin to visualize F-actin in green (**F′′**, **G′′**), anti-acetylated tubulin to visualize nerve tracts in red (**F′′′**, **G′′′**) and their overlap in merged images (**F′′′′**, **G′′′′**). x marks the position of the mouth, I – V denotes the arm pairs in the order they are spatially positioned. Arms are oriented with aboral to the top and distal to the left. White arrowheads in (**A′**) point at the axonal tracts of the interbrachial ganglia extending into individual arm buds. Dashed line in (**B**-**B′′, C**-**C′′**) marks the area of the axial nerve chord. Open arrowheads point at longitudinal muscle fibers, arrows denote transverse muscle fibers. ci, cilia; ep, epithelium; mu, musculature; icm, inner cell mass; su, sucker. Scale bars: 100 μm in (**A**), 10 μm in (**B**)

The subsequent stage is characterized by an elongation of all arms along their PD axes (Fig. 4A), and an increase of cilia on the arms’ aboral surfaces (Fig. 4A′). Since in octopus the arms’ elongation is driven by a concomitant elongation of epithelial cells [22], we compared cell shapes in the epithelium of arms II and IV at stages 21 and 23. At stage 21 epithelial cells on the aboral surface of arm II are oriented at an angle towards the corresponding margins of the arm (Additional file 3A), while at stage 23 elongated rows of cells can be observed, which align in a central region along the arm’s PD axis (Additional file 3A′). In contrast, elongated cells at the proximal base of arm IV are already lined up in central rows along the PD axis at stage 21 (Additional file 3B). At stage 23, most cells in the epithelium of arm bud IV are elongated and oriented along the PD axis (Additional file 3B′).

Except for a central region, cell proliferation at stage 23 is still rather uniform in both arms II and IV (Fig. 4B, C). The central region constitutes the forming neuropil of the future axial nerve cord, which is surrounded by a denser layer of the forming muscle cells with elongated nuclei (Fig. 4B′, C′). In both arms the rudimentary musculature at this stage consists of sporadic individual longitudinal and transverse muscle fibers (Fig. 4B'', C''). Furthermore, the neuropil region in both arms extends almost along the entire length of the arm primordium (Fig. 4B''', C''', B'''', C'''').

### Tissue differentiation

**Fig. 5:**
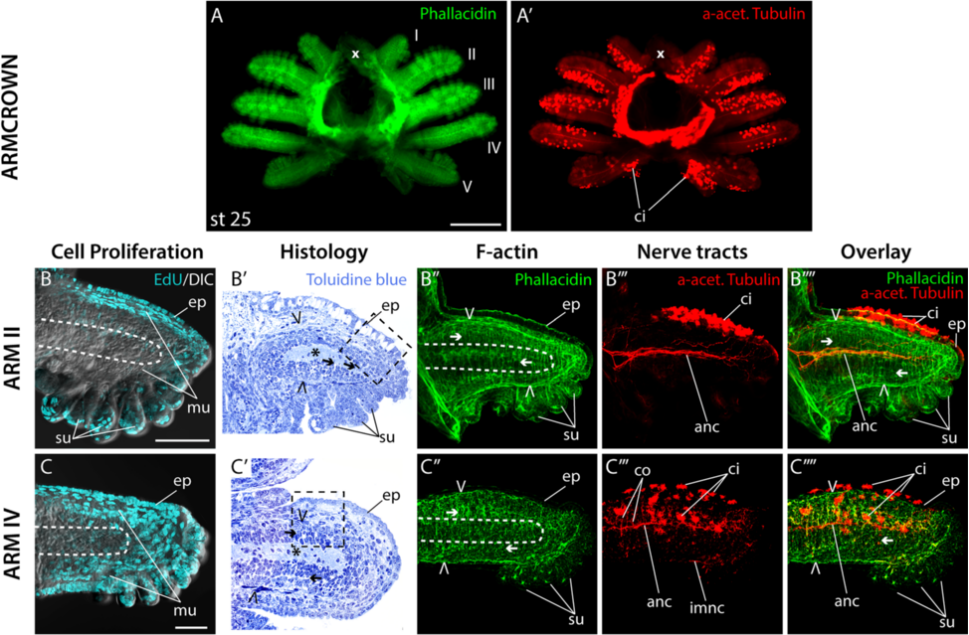
Arm differentiation. (**A**-**A′**) oral view of arm crowns at stage 27 labeled with phallacidin to visualize F-actin in green (**A**) and anti-acetylated tubulin to visualize nerve tracts in red (**A′**). (**B**, **C**) confocal image of frontal sections of arm II (**B**) and arm IV (**C**) treated with EdU to visualize proliferating cell nuclei in cyan, merged with a DIC image of the arms in the same focal plane stacks. (**B′**, **C′**) sagittal histological sections of arms stained with toluidine blue. (**B′′**- **C′′′**) confocal image stacks of arm II (**B′′**- **B′′′′**) and arm IV (**C′′**- **C′′′′**) labeled with phallacidin to visualize F-actin in green (**F′′**, **G′′**), anti-acetylated tubulin to visualize nerve tracts in red (**F′′′**, **G′′′**) and their overlap in merged images (**F′′′′**, **G′′′′**). x marks the position of the mouth, I – V denotes the arm pairs in the order they are spatially positioned. Arms are oriented with aboral to the top and distal to the left. Dashed line in (**B**, **B′′, C**, **C′′**) and asterisk in (**B′**, **C′**) mark the area of the axial nerve chord. Open arrowheads point at longitudinal muscle fibers, arrows denote transverse muscle fibers, white arrowheads indicate emerging connective fibers. Dotted rectangles mark close-up shown in Additional file 4. anc, axial nerve cord; ci, cilia; ep, epithelium; mu, musculature; inmc, intramuscular nerve cord; su, sucker. Scale bars: 100 μm in (**A**), 10 μm in (**B**, **C**)

By stage 25 the central region of the arm crown becomes more restricted, which moves arm pair I closer to each other and towards the mouth. Accordingly, all other arms attain their final position relative to each other and acquire a unique shape and length (Fig. 5A; compare Fig. 4A-A'). During this process the entire arm crown shifts to the anterior region of the head to eventually surround the mouth [7, 9]. Arm pair I remains the shortest, followed by arm pair V, which develops a wider base and grows at an oblique angle relative to the remaining arm pairs. Both arm pairs II and III are rather similar in shape at this stage. Arm pair IV is easily distinguished by its slender shape and its rapid increase in length. Furthermore, the ciliation on the aboral side of all arm pairs becomes localized to arm – specific regions (Fig. 5A′). In particular, the ciliation of arm pairs I, II and V is concentrated to the posterior part of the arms while ciliation of arms III-IV shows a more scattered pattern with a slightly higher density of cilia on the arm’s anterior side.

At stage 25, most cell proliferation becomes localized to the epithelium, a region adjacent to the epithelium, in a central region and the suckers in arm II. Few proliferating cells can also be observed in central regions of the arm (Fig. 5B). The dorsal epithelium in arm bud II consists mostly of large, ovate cells, characterized by a basal nucleus, interjected by interstitial cells. Small, non-secretory, cuboidal cells cover the distal tip, as described in Singley [31] (Fig. 5B′, Additional file 4A). Underneath the epithelium, the layers of longitudinal muscle fibers become more prominent and are partly intertwined with the transverse muscle fibers (Fig. 5B'-B''). The central neuropil region is increasing in size and surrounded by a dense layer of cells with rounded cell nuclei, which Kier [16] identified as neuronal cell bodies (Fig. 5B'). Axon tracts from the axial nerve cord reach the distal tip of the arm (Fig. 5B'''-B''''). In contrast to arm II, cell proliferation becomes most strongly localized to the epithelium, the cell layers adjacent to the distal epithelium and the suckers of arm IV at stage 25. Fewer cells at this stage proliferate in the proximal regions of cells adjacent to the epithelium and in central regions of the arm (Fig. 5C). Furthermore, a single layer of non-secretory cells makes up the epithelium of arm IV (Fig. 5C', Additional file 4B). Underneath the epithelium, longitudinal muscle fibers organized in thick muscle bundles become obvious and are equally intertwined by transverse muscle fibers (Fig. 5C'-5C''). The neuropil area is less prominent than in arm II but is equally surrounded by a dense layer of cells with spherical nuclei (Fig. 5C'). According to Grimaldi et al. [32], these cell bodies surrounding the neuropil constitute differentiating myocytes in the tentacle (arm IV) of the cuttlefish. Here, we consider them as a mixture of differentiating neuronal and muscular cells. The axonal tracts of the axial nerve cord reach throughout the entire length of the arm as well. In addition, intramuscular nerve cords appear on the oral side of arm IV, while the first connective fibers start to project from the axial nerve cord towards the periphery (Fig. 5C'''-C''''). In general, tissue differentiation occurs in a gradual process from the proximal base towards the distal tip in both arms II and IV.

### Tissue end-differentiation

**Fig. 6:**
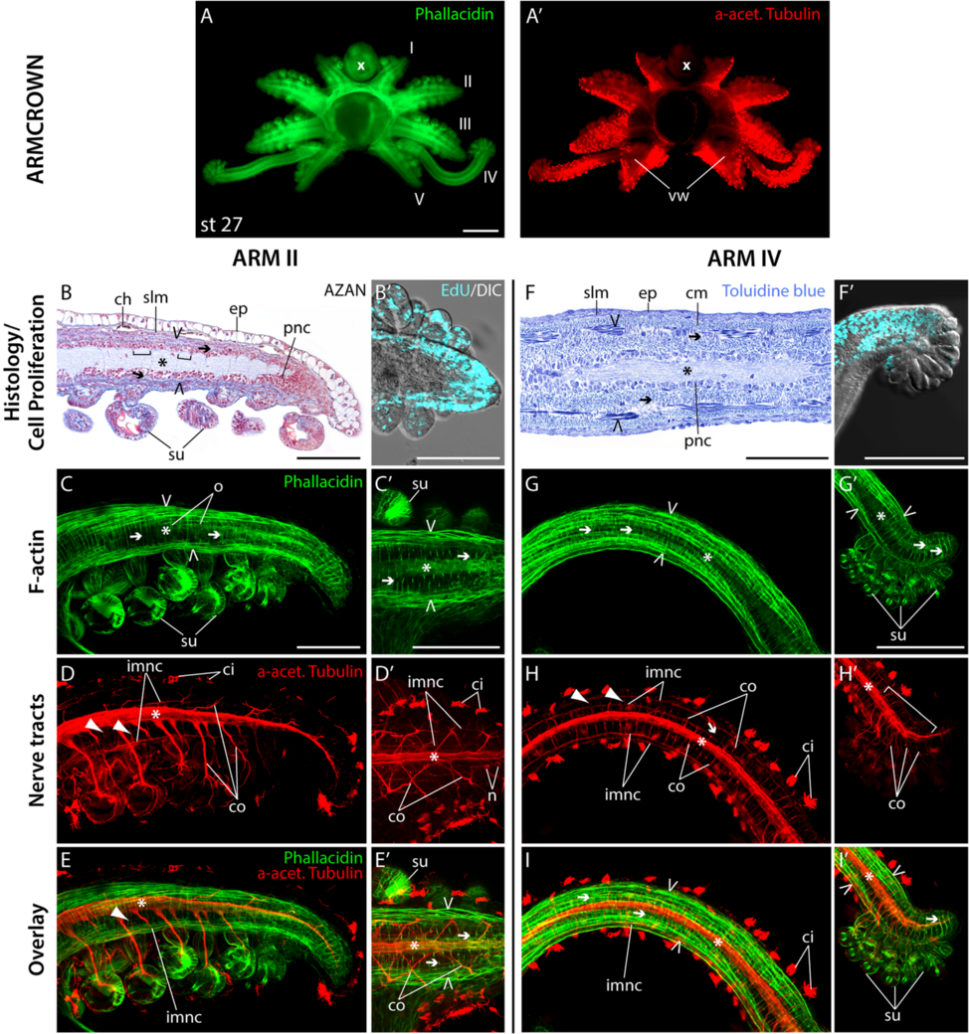
Arm end-differentiation. (**A**-**A′**) oral view of arm crowns at stage 27 labeled with phallacidin to visualize F-actin in green (**A**) and anti-acetylated tubulin to visualize nerve tracts in red (**A′**). (**B**) sagittal histological section of arm II stained with AZAN stain. (**B′**) confocal image of frontal sections of arm II treated with EdU to visualize proliferating cell nuclei in cyan, merged with a DIC image of the arms in the same focal plane stacks. (**C**-**C′**) confocal image stacks of arm II stained for phallacidin to visualize F-actin in green. (**D**-**D′**) confocal image stacks of arm II labeled with anti-acetylated tubulin to visualize nerve tracts in red. (**E**-**E′**) overlap of musculature and nerve tracts in merged images. (**F**) sagittal histological section of arm II stained with Toluidine blue. (**F′**) confocal image of median oral sections of arm IV treated with EdU to visualize proliferating cell nuclei in cyan, merged with a DIC image of the arms in the same focal plane stacks. (**G**-**G′**) confocal image stacks of arm IV stained for phallacidin to visualize F-actin in green. (**H**-**H′**) confocal image stacks of arm IV labeled with anti-acetylated tubulin to visualize nerve tracts in red. (**I**-**I′**) overlap of musculature and nerve tracts in merged images. x marks the position of the mouth, I – V denotes the arm pairs in the order they are spatially positioned. Arms are oriented with aboral to the top and distal to the left. Brackets in (**B**) show the extend of single ganglia, asterisk marks the axial nerve cord, arrow points at transverse muscle fibers, open arrowhead indicates the longitudinal muscle fibers, arrowhead indicates anastomoses. ch, chromatophore; ci, cilia; co, connective fiber; ep, epithelium; imnc, intramuscular nerve cord; m, muscle; o, oblique musculature; pnc, putative neuronal cells; su, sucker; slm, superficial longitudinal muscle; v, vein. Scale bars: 100 μm in (**A**), 10 μm in (**B**-**B, F, F′**, **C′**, **G′**)

From stage 27 to hatching the arm crown differentiates into its final adult-like form. During this time, arm pair III becomes slightly longer than arm pair II and forms a velar web on its posterior side through which it becomes connected to arm pair V (Fig. 6A-A'). Ciliation on the aboral side of the arms further intensifies and remains restricted to the posterior region of arms I and II, while it now covers the entirety of arms III-V (Fig. 6A').

The phase of tissue end-differentiation in arm II is characterized by almost an adult-like maturity (Fig. 6B) and a confinement of cell proliferation to the distal tip (Fig. 6 B'). First chromatophores are formed underneath the epithelium within the dermis of arm II, while an additional superficial-longitudinal muscle layer appears adjacent to the dermis (Fig. 6B). Distinct layers of longitudinal, oblique, and transverse muscle fibers enclose an area of undifferentiated cells adjacent to the neuropil of the axial nerve cord (Fig. 6B, C-C'). The latter is almost devoid of cell bodies and is now comprised of series of ganglia, each of which corresponds to a sucker on the oral side of the arm (Fig. 6B). Connective fibers link the axial nerve cord to the suckers as well as the intramuscular nerve cords within the growing muscle mass. The latter are regularly connected by anastomoses (Fig. 6D-D', E-E'). Similar to arm II, tissue maturity is highly advanced in arm IV and cell proliferation is restricted to the distal portion of arm IV at this stage (Fig. 6F-F'). Furthermore, a superficial and circular muscle layer have formed adjacent to the epithelium in addition to the longitudinal and transverse muscle layer (Fig. 6F, G-G'). However, as opposed to arm II, the axial nerve cord is not organized into a series of ganglia, but consists of a tube-shaped neuropil, which is also almost devoid of cell bodies (Fig. 6F, H). While connective fibers and anastomoses are connecting intramuscular nerve cords to the axial nerve cord and to each other throughout the entire length of arm IV (Fig. 6H, I), an increase in complexity similar to the arm II can only be observed at the very distal tip on the level of the suckers (Fig. 6H', I').

### Formation of the suckers

*E. scolopes* exhibits four rows of typical decabrachian suckers on the arm’s oral surface used for prey handling and egg deposition in the female squid, and more than 32 lines of suckers on the tentacular clubs, which are mostly used for prey capture [33]. Suckers are asymmetrical, stalked, and divided into an infundibulum (attachment face) and an acetabulum (sucker chamber) [34].

**Fig. 7:**
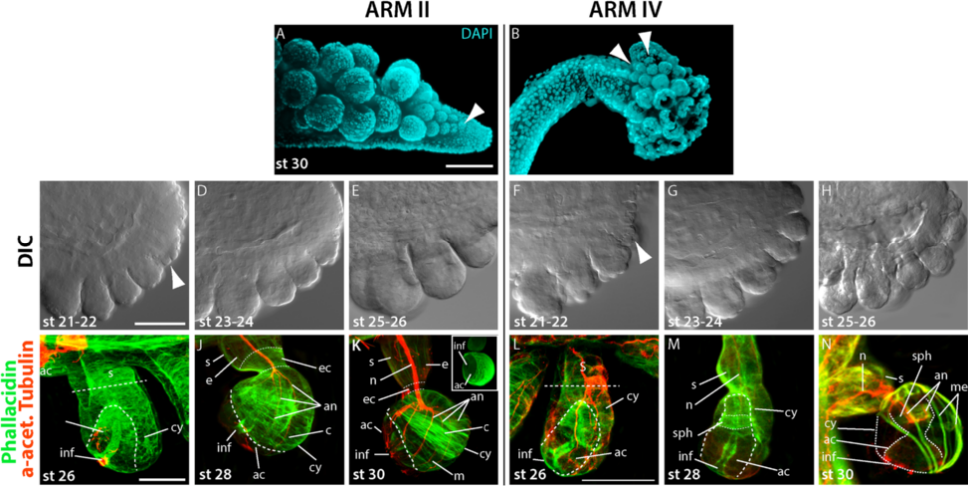
Formation of the suckers. **a**-**b** epifluorescent images of suckers on arm II (**a**) and arm IV (**b**) at stage 30 stained for DAPI to visualize cell nuclei in cyan. **c**-**h** DIC images of suckers on arm II (**c-e**) and arm IV (**f-h**) at stages indicated in the bottom left corner. Arms are oriented with distal to the left, aboral to the top. **i**-**n** merged confocal image stacks of individual suckers on arm II (**i**-**k**) and arm IV (**l**-**n**) labeled with phallacidin to visualize F-actin in green and for acetylated tubulin to visualize the nerve tracts in red at stages indicated in the bottom left corner. Inset in (**k**) shows an epifluorescent image of a sucker of at stage 30 stained for phallacidin. Arrowheads point at area of sucker formation. ac, acetabulum; an, acetabular nerve; c, circular muscle; cn, connective nerve; e, extrinsic muscle; ec, extrinsic circular muscle; cy, cylinder, inf, infundibulum; me, meridional muscle; s, stalk; sph, sphincter muscle. Scale bars: 100 μm in **a**, 50 μm in **c**, and 10 μm in **i**, **l**

**Fig. 8:**
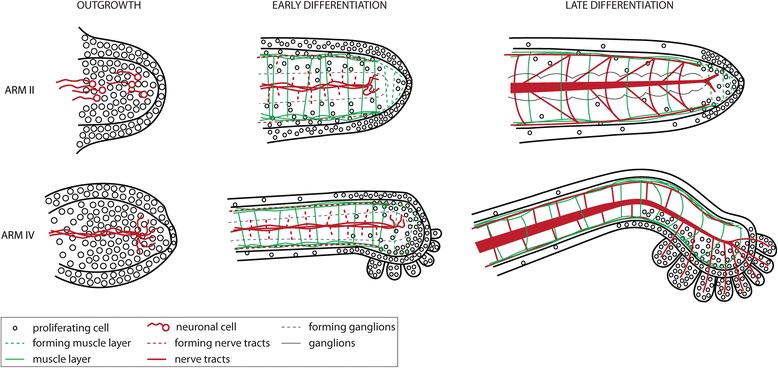
Summary of the major events of arm formation. The development of the *E. scolopes* appendages can be divided into three distinct phases which show temporal and spatial differences between sessile arms and tentacles

During embryonic development suckers appear as rounded papillae on the distal rim of the arm’s oral surface and new suckers are added in this area throughout the embryo’s development. On arm II this mechanism produces suckers in a constant manner in which suckers are added one at the time, increase in size, and form a double, triple, and finally quadruple row while the arm extends along its PD axis (Figs. 7a and 8). Conversely, in arm IV multiple suckers are formed simultaneously but do not organize into well-defined rows (Fig. 7b).

Early sucker primordia consist of a mesodermal cell mass surrounded by a simple epithelium (Fig. 7c-h). Starting at stage 25 the largest suckers of both arm II and IV show first signs of differentiation at which suckers on arm II are generally larger than those on arm IV (Fig. 7e, h). At stage 26, a short stalk can clearly be distinguished from the ovate future cylinder, which contains the primordial acetabulum and an infundibulum in both arms II and IV (Fig. 7i, l). Within only a few days of development, by stage 28, the suckers on arm II and IV have matured considerably and show first structural differences (Fig. 7j, m). Suckers on both arms consist of a muscular stalk with a constricted end, which attaches to the cylinder containing the acetabulum. While the extrinsic musculature of suckers on arm IV does not show any specializations yet, the constriction of suckers on arm II consists of a defined layer of extrinsic circular muscle fibers. Unlike the suckers on arm II, the acetabulum of the suckers on arm IV show a well-formed sphincter muscle separating the acetabular roof from the rest of the structure. Both sucker types are connected to the axial nerve cord by a connective nerve fiber, which divides into several acetabular nerve fibers at this stage. Shortly before hatching the cylinder of suckers on arm II consists mostly of circular muscle fibers and does not completely envelope the acetabulum consisting of circular and meridional muscle fibers. The infundibulum is rather small and a dense network of nerves appears at its rim (Fig. 7k). Conversely, the cylinder of the suckers on arm IV is mostly made up of meridional muscle layers and is completely surrounded by the acetabulum. The sphincter muscle at the base of the acetabulum becomes even more apparent and a dense network of nerves innervates the rim of the infundibulum’s broad opening, similar to what is observed in suckers of arm II (Fig. 7n).

## Discussion

The embryonic development of the *E. scolopes* arm crown is a dynamic process during which an adult-like structure is established. With the exception of the hectocotylus, which is modified during sexual maturation of the juvenile male squid [35], the arms and tentacles are fully functional at hatching stage [36]. This stands in contrast to other decabrachian species, that produce small eggs and immature, paralarval hatchlings, in which the tentacles and associated adult-like prey capture behaviors mature during post-hatching stages (e.g., *Sepioteuthis lessoniana, Loligo vulgaris*) [37, 38].

### Early outgrowth and elongation of the *E. scolopes* appendages

Similar to octopus appendage formation, the *E. scolopes* arm crown is initiated as an epithelial thickening, which divides into prospective arm fields consisting of small, condensed, epithelial cells [22]. During the subsequent phase of arm outgrowth, spherical arm bulges are established by means of isotropic cell proliferation, which consist of a histologically not discernable cell mass surrounded by an epithelium. The elongation of the arm bulge along its PD axis marks the onset of the differentiation into mature tissue types (Fig. 8). This particular subdivision into an initiation by setting apart a subset of progenitor cells, growth through cell proliferation, morphogenesis and differentiation is not necessarily specific to cephalopod appendage development but lies at the very basis of organ formation [39, 40]. It is therefore not surprising, that appendage development in a diverse range of animal phyla seem to follow this pattern [41–45].

However, one defining characteristic of appendage development constitutes an elongation along the PD axis during the phase of morphogenesis. In *E. scolopes* we observed epithelial cell re-arrangements during this phase, which are especially pronounced in arm IV and may account for the rapid elongation of the future retractile tentacle. Generally, epithelial cell dynamics, such as epithelial thickenings and epithelial cell shape changes seem to be common phenomena of appendage outgrowth and elongation throughout the animal kingdom. For instance, the tentacle precursors of the sea anemone *Nematostella vectensis* arise from thickened epithelial placodes within the oral ectoderm, the outgrowth and elongation of which are correlated with oriented epithelial cell rearrangements [44]. Similarly, the appendages of the adult fruit fly *Drosophila melanogaster* originate from clusters of epithelial cells, which invaginate and proliferate to form the imaginal wing and leg discs [45, 46]. The elongation of these appendages and their reorganization into adult shape are achieved by cell shape changes in both legs and wings [45–49]. Furthermore, the outgrowth of the zebrafish’s (*Danio rerio*) median and pectoral fins is achieved by changes of epithelial cell shapes from elongated to round [50]. Similar cellular dynamics were even observed during the formation and elongation of vertebrate epithelial appendages, such as feathers, scales, hair, claws, and teeth [50–53]. Recent studies on the embryonic formation of octopus appendages have shown that actin-mediated epithelial cell shape changes (i.e., cell elongation and their alignment along the PD axis) are also crucial for the elongation of appendages in a cephalopod [22]. Further studies on cell-cell interaction and cell proliferation will help confirm whether epithelial cell shape changes are equally involved in the elongation process of the *E. scolopes* appendages or show unrelated cellular or morphogenetic functions (e.g., cell migration, increase in the arms’ thickness).

Therefore, an elongation along the PD axis seems to be a shared characteristic of appendage formation, even though the mechanisms by which it is achieved may vary depending on animal phyla and appendage type. This similarity may reflect a common need of an appendage to extend beyond the primary body axis to perform its locomotory or sensory purpose. While this observation does not imply any evolutionary significance per se, it may imply that a shared molecular mechanism exists, which drives the outgrowth and PD elongation of appendages regardless of their function and identity. Considering the presumed evolutionary origin of the cephalopod arm crown from the ventral molluscan foot it is rather surprising that arms are formed as individual entities rather than being sculpted from an existing muscular foot by programmed cell death, similar to the formation of digits in vertebrates [54]. It would be interesting to investigate whether an existing appendage-specific program involved in the PD outgrowth has been recruited into novel locations within the molluscan foot to initiate appendage outgrowth in cephalopods.

### Differentiation of the *E. scolopes* appendages

Histologically discernible tissue layers appear as soon as the arm primordia start to elongate. In both arm pairs, II and IV, the onset of differentiation is characterized by the formation of distinct muscle layers underneath the epithelium and a neuropil within the cell mass of the axial nerve cord (Additional file 5A). While the early set-up of arms II and IV looks rather similar, at differentiation stage their morphology shows differences on most tissue levels (Fig. 8). In particular, the epithelium of arm II is comprised of large secretory ovate cells while the arm IV is covered by a simple, single-layered epithelium of non-secretory cells (Additional file 5B). Furthermore, the muscle arrangement and types differ between arm II and arm IV, in which the organization of the longitudinal muscle fibers into distinct bundles is the most conspicuous feature (Additional file 5C). Finally, the axial nerve cord is organized into a series of ganglia connected by nerve fibers in arm II, whereas in arm IV a ganglionic organization can only be observed at the distal tip of the arm on the level of its suckers. Furthermore, both spatial and temporal differences exist in the maturation of tissue types within each arm and between both arms: (i) tissue maturation begins proximally and gradually continues towards the distal tip, (ii) neuronal cells appear before mature muscle cells are visible, and (iii) arm IV generally shows a higher cellular complexity at younger stages, most likely due to an expedited growth rate.

### Differentiation of the musculature

During the embryonic formation of the octopus’ (*Octopus vulgaris*) arms, only transverse and longitudinal muscle fibers are formed, whereas the maturation of all other muscle types is postponed to paralaval post-hatching stages [22]. In *E. scolopes*, transverse and longitudinal muscle fibers are also the first muscle types to appear at the onset of tissue differentiation, and they remain the most prominent muscle layers in both arms II and IV until shortly before hatching. At this time, additional muscle layers become discernable, which include the superficial longitudinal and rudimentary oblique muscle layers in arm II and the superficial longitudinal and circular muscle layers in arm IV, respectively. These differences in muscle maturity at the time of hatching reflect the animals’ species-specific post-hatching life styles: while the octopus paralarva undergoes a pelagic phase before settling to the adult benthic lifestyle, the *E. scolopes* paralarva hatches as a fully functional mini-adult [36].

Another interesting feature of the *E. scolopes* musculature concerns their prey capture behavior right after hatching. Unlike the tentacles of both decabrachian *Sepiteuthis lessoniana* and *Sepia officinalis*, which only become functional during post-hatching stages, *E. scolopes* tentacles are fully functional after hatching [36]. In *S. lessoniana* functionality of the tentacles relies on the transition of the musculature’s striation pattern from oblique to transverse during post-hatching stages [20]. Conversely, in *S. officinalis* cross-striated muscle fibers already exist at the time of hatching, and the tentacles’ function may depend on either the maturation of the muscle innervation or the correct ratio of smooth-like to striated muscle fibers [55, 56]. Studies on the ultrastructural composition of the tentacles’ musculature in *E. scolopes* may therefore give further insight into the diversification of the cephalopod musculature within these specialized appendages.

### Differentiation of the nervous and sensory system

During octopus arm initiation, neuroblast cells first ingress from the ectoderm into the early limb as soon as the arm field is established [22, 57]. Therefore, immature neuronal precursor cells are likely already present during the early phase of limb outgrowth of the *E. scolopes* appendages. However, maturing neuronal cells only become histologically recognizable as soon as a spherical bulge has formed. These cells project nerve fibers in distinct clusters towards the proximal base of the arm and connect with the axonal projections of the remaining arms in the interbrachial connective. While insect motor neurons as well as vertebrate and annelid sensory and motor neurons innervate appendages by axons that grow into the limb bud from the central nervous system or adjacent ganglia [43, 58–60], the situation observed during the early formation of the *E. scolopes* brachial nervous system is reminiscent of sensory neuron development in insect appendages. In insects maturing neurons appear in the early distal limb bud and project their axons proximally towards the central nervous system. These so-called pioneer neurons act as stepping-stones that lead the path for all later appearing sensory neurons [61–64]. Since this initial observation was made, pioneer neurons have been found to be essential for axonal guidance in nervous cells regardless of their type in a variety of species [65]. Whether the distinct patches of neuronal cells observed in *E. scolopes* include pioneer neurons acting in a similar way is a compelling question that will have to be resolved. In general, pioneer neurons are known to be involved in the early formation of the larval central nervous system in lophotrochozoa but have not yet been reported during the formation of the peripheral or definitive adult nervous system [66].

After a first nerve strand is established, diffuse axonal extensions become visible at the distal tip of the arm, the origin of which could not be determined in this study. On the one hand new pioneer neurons may mature distally and project their axons proximally, extending the nerve strand in a stepwise manner similar to the process observed in locust appendages [63]. Conversely, precursor cells of motor or sensory neurons could proliferate proximally or distally and extend their axons towards the tip once they mature.

Additional elements of the arm’s nervous system appear at the onset of differentiation and include the intramuscular nerve cords, connective fibers, and anastomoses. While connective fibers and anastomoses seem to extend from the axial nerve cord, the six intramuscular nerve fibers appear independently as axonal projections close to the distal tip, similar to the early axial nerve cord. In general, both arm pairs II and IV show a similar nervous system arrangement, at which the lack of ganglia in arm IV leads to a ladder-like structure where no suckers are present.

Finally, at stage 21, ciliated cells appear on the aboral epithelium in an arm specific pattern. These cells cover the entire posterior side of arm I and II, and most of the distal surface of arms III, IV, and V. According to Arnold and Williams-Arnold [62], these paddle-shaped ciliated cells may create a current in the chorionic fluid, which causes the embryo to rotate. This interpretation would explain the position of the cilia on the individual arms, which would contribute in a rotation of the embryo along its DV axis. However, recent studies have shown that some of these ciliated cells are in fact ionocytes that are responsible for ion regulation during the early stages of embryogenesis [67–69].

### Differentiation of the suckers

*E. scolopes* suckers are oral appendages that appear as rounded papillae on the distal end of the arm. While suckers on arm II become organized into four distinct rows along the entire length of the arm, suckers on arm IV remain confined to the distal tip and are organized in a less defined pattern. Even though early differentiation seems rather similar, the suckers on arm II differ from the suckers on arm IV in their overall shape, muscle fiber composition, and size at hatching stage. These differences represent adaptations to the respective arm’s specific function (manipulation versus prey capture). For instance small suckers have been shown to produce greater pressure differentials in relation to the surrounding water at higher depth [70]. Therefore, the size of the suckers on the retractile tentacles may be reduced in order to securely retain elusive prey. In comparison to other sepiolid species the *E. scolopes* sucker development is most similar to that of *Rossia macrosoma*, as previously described by Nolte and Fioroni [34]. In both sepiolids suckers are highly differentiated at hatching stage - a typical feature of cephalopod species that produce large, yolky eggs. However, the definitive number of suckers has not been established yet.

### Arm homologies

Similarities in the formation of arm pairs II and IV seem to exist mostly during the early outgrowth phases of the appendages. Major differences in arm formation include an expedite growth rate, a variation in the muscle composition and the restriction of suckers to the distal end of arm IV. In comparison to octopod arm development it seems more likely that the octopus arms correspond to the *E. scolopes* sessile arms and that the tentacles represent a modification thereof. However, it is interesting that arm loss in octopus has already become manifested during embryonic development and a rudimentary fifth arm field could not even be observed during early arm field appearance [71]. This stands in contrast to the development of the arm crown in the pygmy squid *Idiosepius*, in which all five arm fields are present during early arm formation, but arm pair IV does not elongate until after hatching [72]. Even though based on the results obtained from this work it is not possible to verify the current hypothesis of arm homology (Additional file 1) it appears that arm pair IV is frequently subject to diversification within the class of cephalopods and may therefore be more prone to loss. The diversifications and loss of these serially homologous appendages would be a fascinating topic to investigate on a molecular level. For instance, shifts in *Hox* gene expression domains play an important role in both, change of morphology and number of appendages in insects [39]. Lee et al. [71] showed that the identity of each of the *E. scolopes* appendages may be specified by a unique combination of *Hox* gene orthologues. Therefore, comparing the expression of *Hox* genes or similarly conserved regulatory gene networks between arm types and cephalopod orders may help to conclusively resolve this question. Furthermore, our results raise the question whether gene regulatory pathways involved in early PD outgrowth and patterning have been recruited to the ventral foot region of an ancestral cephalopod and initiated outgrowth of individual appendage entities. Studying genes and gene regulatory pathways involved in these events may give us as a new perspective on the evolution of animal appendages.

## Conclusion

The formation of the *E. scolopes* arm crown is a dynamic process divided into distinct phases. These include (i) the appearance of the armcrown, (ii) separation into arm fields, (iii) arm outgrowth, (iv) elongation along the PD axis and initiation of differentiation, and (v) tissue (end-) differentiation. The early outgrowth and elongation of the arms is characterized by an isotropic cell proliferation and the onset of tissue differentiation. While early outgrowth is similar in all arms, subsequent differentiation of the appendages shows differences at most tissue levels. Generally, arm IV shows higher complexity at younger stages and different muscular and nervous tissue composition. However, tissues differentiate in a gradient from proximal to distal, whereas cell proliferation becomes restricted to the distal-most end of both arms. Similarities to appendage formation of other well-studied model organisms seem to exist and raise the question whether these similarities reflect the parallel recruitment of similar molecular patterning modes.

## Methods

### Animals

Adult *Euprymna scolopes* specimens were collected at nighttime along the shores of Hawaii Kai and Kaneohe bay, Oahu, Hawaii. Males and females were kept separately in 140 × 100 × 90 cm fiberglass tanks with a flow through system and fed with live shrimp (*Palaemon debilis*). Each female was allowed to mate for three consecutive days every other week and was provided with PVC half pipes for spawning. Egg clutches were carefully removed from the substrate, transferred into glass bowls of 20 μm filtered seawater (FSW) and incubated at 24 °C with daily seawater changes. Squid embryos were manually removed from the outer capsule and jelly coat using watchmaker’s forceps and staged according to Lee et al. [25].

### Fixation

Embryos contained within the chorion were relaxed for 30 min in a 1:1 dilution of 0.37 M MgCl_2_:FSW and prefixed for one hour in a 4 % formaldehyde solution, made freshly by dilution of paraformaldehyde (Electron Microscopy Sciences, Hatfield, PA, USA) in 0.2 μm FSW at room temperature. After five FSW rinses, embryos were manually dechorionated and post-fixed according to one of following fixation methods: Embryos to be used for histological sectioning were fixed in Bouin’s fluid for two days at room temperature, washed 5 times for 5 min in marine PBS (mPBS; 50 mM sodium phosphate buffer with 0.45 M NaCl; pH 7.4) and stored in 70 % ethanol in mPBS at 4 °C until analysis. For antibody labeling of early stages (stages 17 – 20) embryos were fixed over night at 4 °C in 4.2 % paraformaldehyde in PBS containing 0.1 M HEPES (4-(2-hydroxyethyl)-1-piperazineethanesulfonic acid, pH 6.9), 50 μM EGTA (Ethylene glycol-bis (2-aminoethylether)-N,N,N′,N′-tetraacetic acid, pH 8–9), 5 μM MgSO_4_, 0.4 M Dextrose, and 4 % Triton X-100. Animals were rinsed several times in mPBT (mPBS + 1 % Triton X-100) and immediately processed.

### Histology

Samples were dehydrated in a graded series of ethanol (80, 90, 95, 100 %), embedded in paraffin and cut with a Reichert-Jung rotational microtome in 7 μm sections. The sections were stained with azocarmine-anilin blue (AZAN) according to the Heidenhain staining protocol [73]. For semi-thin sections embryos were embedded in Araldite (Sigma-Aldrich, St. Louis, MO, USA) after dehydration and sectioned using a Reichert-Jung Ultracut E rotational microtome and a HistoJumbo diamond knife into consecutive series of 1 μm sections. Samples were stained with toluidine blue in 1 % Borax [74] and sealed with Araldite.

### Immunolabeling

Embryos were permeabilized with mPBT at 4 °C overnight. Non-specific binding sites were blocked with blocking solution consisting of mPBT + 10 % normal heat-inactivated goat serum (Sigma-Aldrich, St. Louis, MO, USA) for 2 h at room temperature. Subsequently, embryos were incubated in primary antibody in blocking solution over two nights at 4 °C. After extensive washes with mPBS for at least 4 h at room temperature animals were incubated in secondary antibody, 1:1000 TO-PRO-3 (Life technologies, Carlsbad, CA, USA), and 1:200 BODIPY FL-phallacidin (Life technologies, Carlsbad, CA, USA) or Alexa Fluor 488-phalloidin (Life technologies, Carlsbad, CA, USA) in mPBS + 10 % normal heat-inactivated goat serum for 2 – 3 days at 4 °C. Following several mPBS washes animals were cleared in 70 % glycerol over night at 4 °C and mounted for analysis. Early stage embryos (stage 17–20) were incubated with mouse-anti-histone H1 (F152.C25.WJJ, Millipore) (1:500) and later stage embryos (stage 21–30) with mouse-anti-acetylated tubulin (6-11B-1; Sigma-Aldrich, St. Louis, MO, USA) (1:1000) as primary antibody. Secondary antibodies used were either donkey-anti-mouse Alexa Fluor 546 (Life technologies, Carlsbad, CA, USA) (1:400), or goat-anti-mouse Alexa Fluor 568 (Life technologies, Carlsbad, CA, USA) (1:500).

### EdU labeling

DNA synthesis in proliferating cells was detected using the Click-It EdU Alexa Fluor 488 imaging kit (Life technologies, Carlsbad, CA, USA). Embryos stage 18–30 were incubated in 0.3 μM EdU for 1 h, and relaxed and fixed as described above. After a few rinses in mPBS animals were incubated in mPBT for 2 h at room temperature or overnight at 4 °C. Subsequently, embryos were washed 5 times for 5 min in mPBS + 3 % BSA (Bovine Serum Albumin, pH 7.4) and incubated in the reaction cocktail (mixed according to the manufacturer’s protocol) for 30 min at room temperature. Animals were washed 5 times for 5 min in mPBS, cleared in 70 % glycerol over night at 4 °C and mounted for analysis.

### Microscopy

Immunolabeled overview preparations as well as histological preparations were viewed, analyzed and documented using either an Axioskop 2 compound light microscope (Zeiss) with a stem-mounted SpotFlex digital camera (Diagnostic Instruments) or an Axio Imager.A1 compound light microscope (Zeiss) with a ProgRes C14 plus digital camera (Jenoptik, Germany). In order to improve the depth of field, selectively focused images were stacked and combined using Helicon focus 4.2.7 software (Helicon Soft Ltd.) in some instances. Confocal imaging was performed using either a LSM 710 (Zeiss) or a CLSM 2 (Leica) confocal microscope, and 3D images were created using ImageJ (NIH).

## Additional files

Additional file 1: The current hypothesis on arm homologies between cephalopods. Embryonic and comparative morphological data suggests that the second arm pair was lost in the octobrachian cephalopods and modified in *Vampyroteuthis*, while the fourth arm pair was modified into retractile tentacles in decabrachian cephalopods. (PNG 1643 kb) Additional file 2: Close-up of arm fields II and IV at stage 19. Arm field consist of a cluster of small epithelial cells. White boarders mark the outline of arm field. Scale bar: 50 μm. (PNG 1469 kb) Additional file 3: Epithelial cell shapes on the aboral surface of the arms during phases of arm outgrowth and elongation. Confocal image stacks of surface of arm II (A- A′) and arm IV (B′- B′) stained for phallacidin to visualize F-actin. Red line outlines elongated cells oriented along the PD axes of the arms. Scale bars: 50 μm. (PNG 3450 kb) Additional file 4: Close up of dotted section in Fig. 5B’ and C’. (A) Epithelium and adjacent tissue layers of arm II (B) epithelium and adjacent tissue layers of arm IV. Parenthesis marks cell area surrounding axial nerve cord, asterisk denotes the axial nerve cord. c, cilia; cc, cuboidal cell; e, epithelium; ic, interstitial cell; oc, ovate cell. Scale bar: 10 μm. (PNG 2694 kb) Additional file 5: Comparison of arm II (A-C) and arm IV (D–F) development. (A, B, D, E) semi-thin, transverse histological sections from the proximal region of the arm stained with toluidine blue. (C, D) transverse histological sections from the proximal region of the arm stained with AZAN. c, circular muscle; ch, chromatophore; d, dermis; ep, epithelium; fml, future muscle layer; h, helical muscle; lm, longitudinal muscle; ml, muscle layer; anc, axial nerve cord; o, oblique muscle; pnc, putative neuronal cells; slm, superficial longitudinal muscle; s, sucker; tm, transverse muscle; tr, trabeculae; v, vein. Arrow marks elongated myoblast cells within the tissue, arrowhead points out spherical putative neuronal cells enveloping the axial nerve cord. Scale bar: 100 μm. (PNG 6581 kb)

## Abbreviations

ac: Acetabulum
an: Acetabular nerve
anc: Axial nerve cord
ar: Artery
c: Cilia
cc: Cuboidal cell
ch: Chromatophore
cm: Circular muscle
cn: Connective nerve
cy: Cylinder
d: Dermis
e: Extrinsic muscle
e: Eye
ec: Extrinsic circular muscle
ep: Epithelium
fi: Fin
fml: Future muscle layer
fu: Funnel
h: Helical muscle
ic: Interstitial cell
icm: Inner cell mass
inf: Infundibulum
inmc: Intramuscular nerve cord
lm: Longitudinal muscle
m: Mantle
me: Meridional muscle
ml: Muscle layer
mu: Musculature
o: Oblique muscle
oc: Ovate cell
pnc: Putative neuronal cells
s: Stalk
slm: Superficial longitudinal muscle
sph: Sphincter muscle
su: Sucker
tm: Transverse muscle
tr: Trabeculae
v: Vein
