# Supplementary figures and images for: The cephalopod arm crown: appendage formation and differentiation in the Hawaiian bobtail squid Euprymna scolopes
Source: Front Zool. 2016 Sep 29;13:44. doi: 10.1186/s12983-016-0175-8 (PMC5041568; doi:10.1186/s12983-016-0175-8)

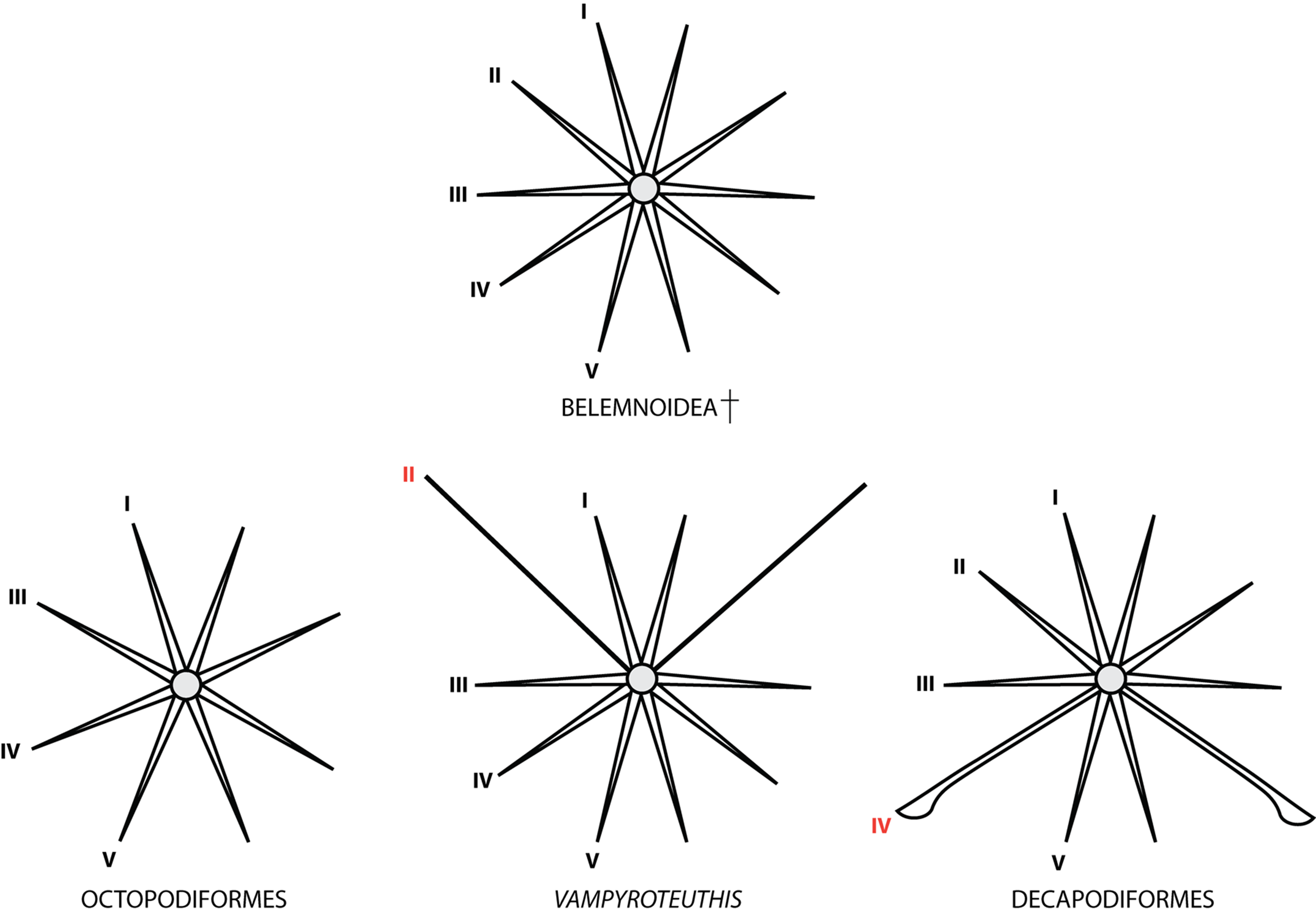

Supplement: Additional file 1: — The current hypothesis on arm homologies between cephalopods. Embryonic and comparative morphological data suggests that the second arm pair was lost in the octobrachian cephalopods and modified in Vampyroteuthis, while the fourth arm pair was modified into retractile tentacles in decabrachian cephalopods. (PNG 1643 kb) [file 12983_2016_175_MOESM1_ESM.png]

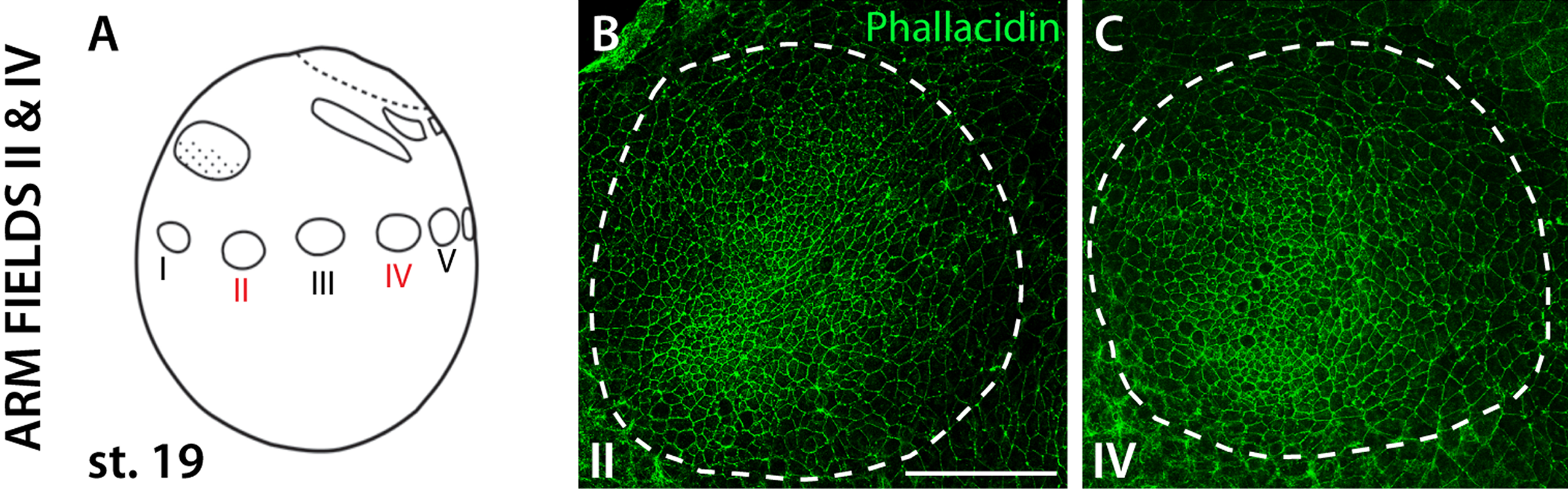

Supplement: Additional file 2: — Close-up of arm fields II and IV at stage 19. Arm field consist of a cluster of small epithelial cells. White boarders mark the outline of arm field. Scale bar: 50 μm. (PNG 1469 kb) [file 12983_2016_175_MOESM2_ESM.png]

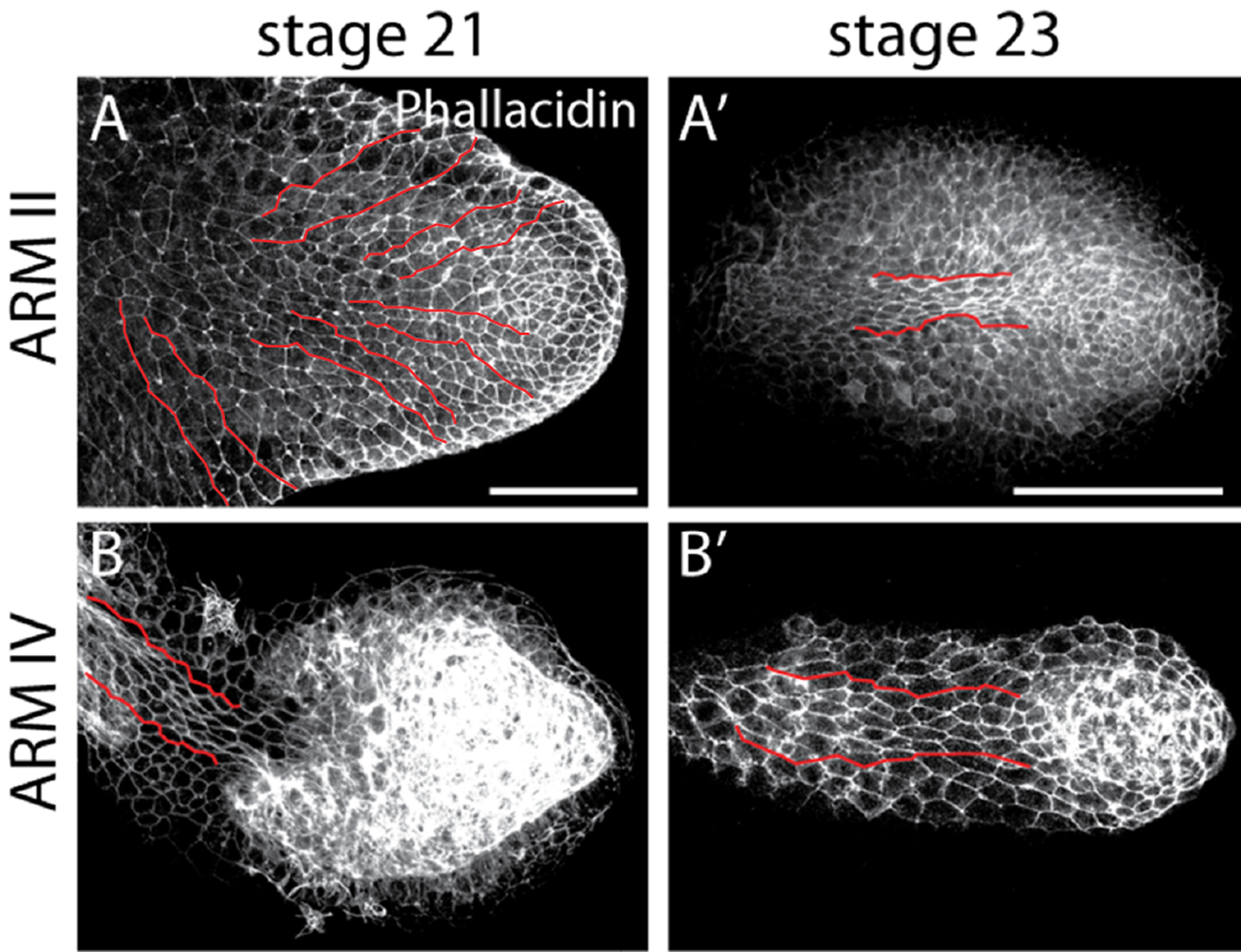

Supplement: Additional file 3: — Epithelial cell shapes on the aboral surface of the arms during phases of arm outgrowth and elongation. Confocal image stacks of surface of arm II (A- A′) and arm IV (B′- B′) stained for phallacidin to visualize F-actin. Red line outlines elongated cells oriented along the PD axes of the arms. Scale bars: 50 μm. (PNG 3450 kb) [file 12983_2016_175_MOESM3_ESM.png]

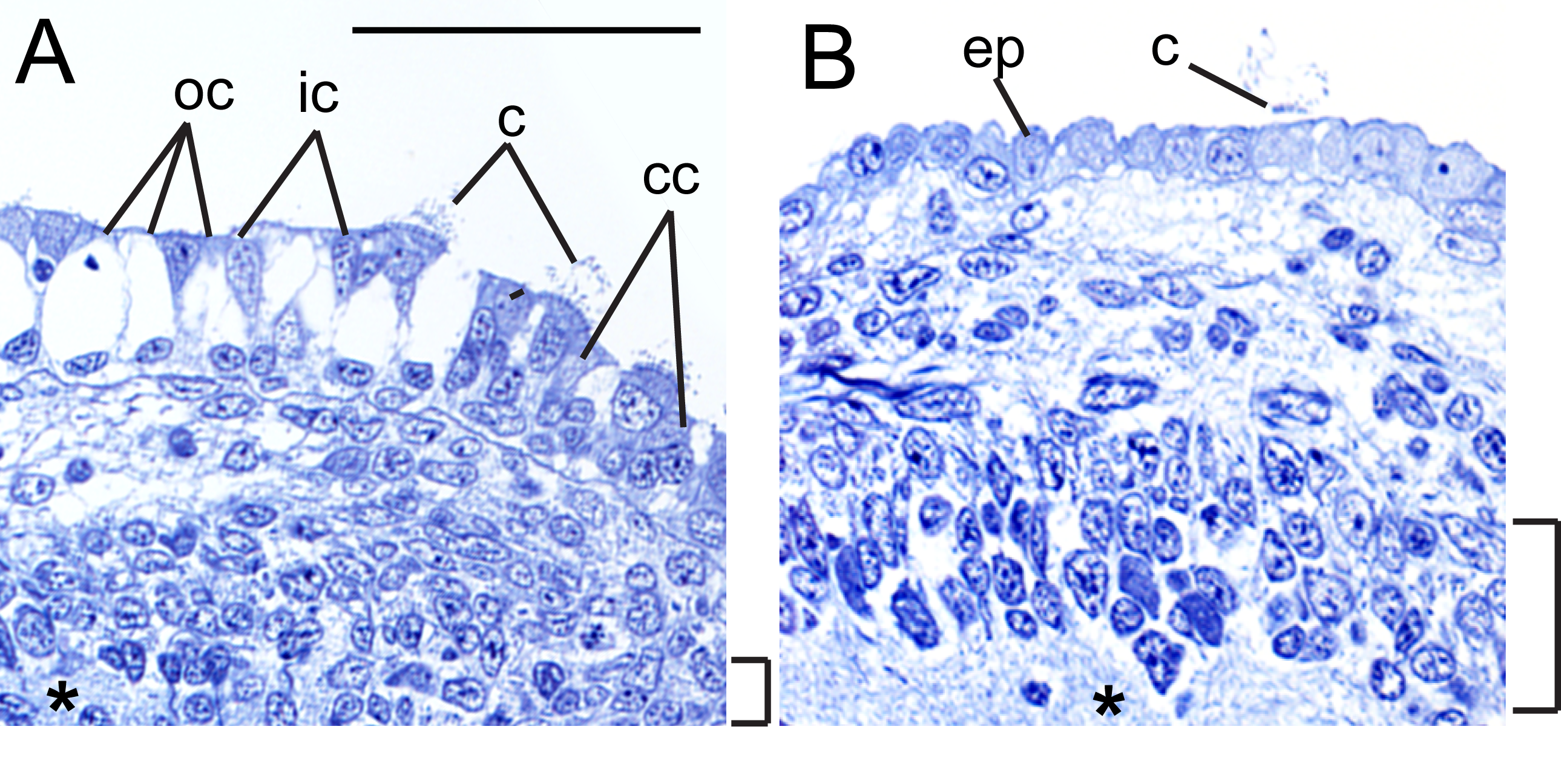

Supplement: Additional file 4: — Close up of dotted section in Fig. 5B’ and C’. (A) Epithelium and adjacent tissue layers of arm II (B) epithelium and adjacent tissue layers of arm IV. Parenthesis marks cell area surrounding axial nerve cord, asterisk denotes the axial nerve cord. c, cilia; cc, cuboidal cell; e, epithelium; ic, interstitial cell; oc, ovate cell. Scale bar: 10 μm. (PNG 2694 kb) [file 12983_2016_175_MOESM4_ESM.png]

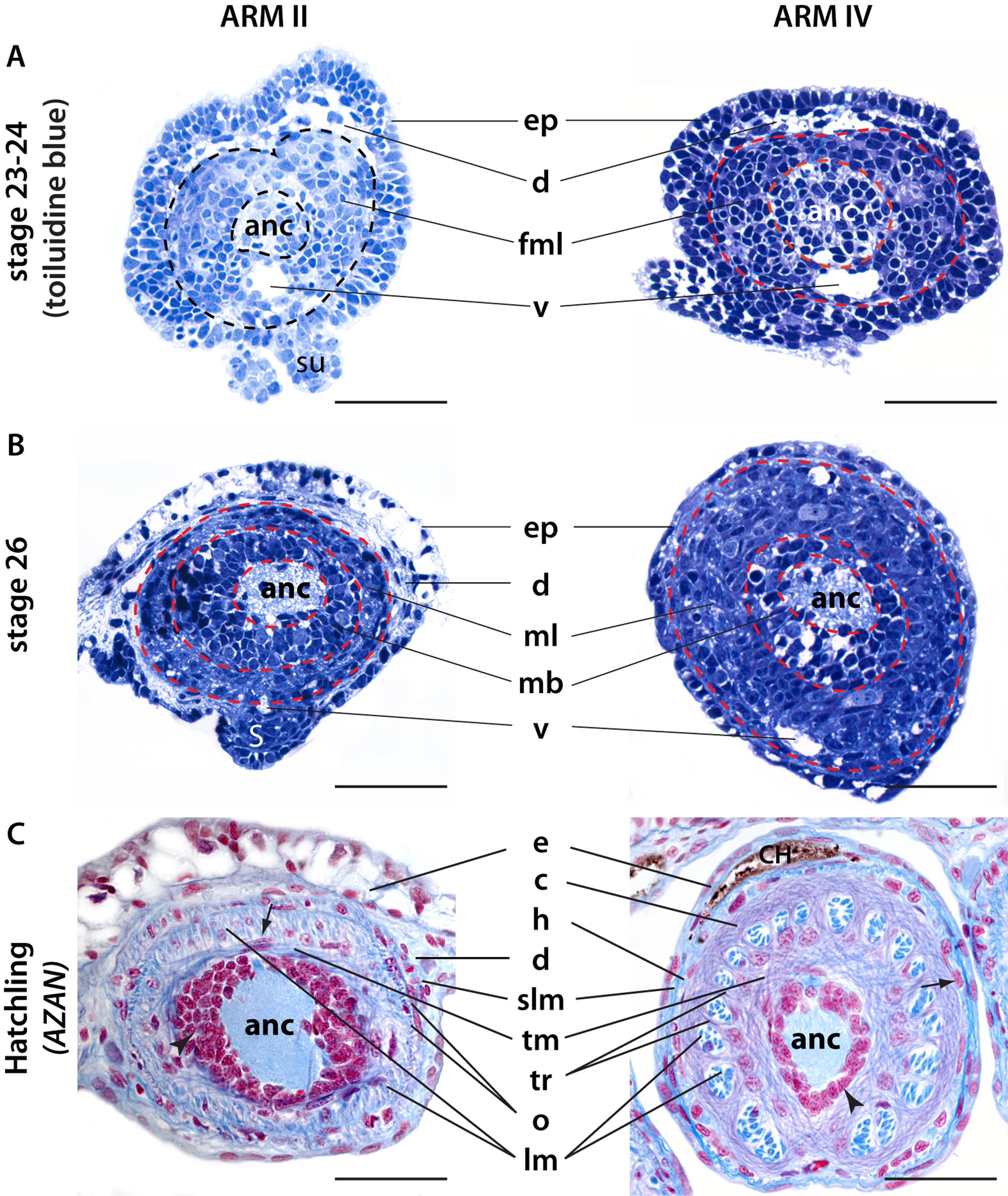

Supplement: Additional file 5: — Comparison of arm II (A-C) and arm IV (D–F) development. (A, B, D, E) semi-thin, transverse histological sections from the proximal region of the arm stained with toluidine blue. (C, D) transverse histological sections from the proximal region of the arm stained with AZAN. c, circular muscle; ch, chromatophore; d, dermis; ep, epithelium; fml, future muscle layer; h, helical muscle; lm, longitudinal muscle; ml, muscle layer; anc, axial nerve cord; o, oblique muscle; pnc, putative neuronal cells; slm, superficial longitudinal muscle; s, sucker; tm, transverse muscle; tr, trabeculae; v, vein. Arrow marks elongated myoblast cells within the tissue, arrowhead points out spherical putative neuronal cells enveloping the axial nerve cord. Scale bar: 100 μm. (PNG 6581 kb) [file 12983_2016_175_MOESM5_ESM.png]
